# Intestinal fungi biogeography, succession and its association with diarrhea in pigs

**DOI:** 10.1186/s40104-025-01206-9

**Published:** 2025-06-04

**Authors:** Ruochen Ren, Xiaojun Zhang, Fangfang Lou, Yang Li, Lingyan Ma, Yingping Xiao, Qu Chen, Yang Wen, Wentao Lyu

**Affiliations:** 1 State Key Laboratory for Managing Biotic and Chemical Threats to the Quality and Safety of Agro-Products, Institute of Agro-Product Safety and Nutrition, Zhejiang Academy of Agricultural Sciences, No.298 Desheng Middle Road, Shangcheng District, Hangzhou, 310021 China; 2 Institute of Animal Husbandry and Veterinary Medicine, Jinhua Academy of Agricultural Sciences, Jinhua, 321017 China; 3 Zhejiang MEBOLO Biotechnology Co., Ltd., Jinhua, 321016 China

**Keywords:** Diarrhea, Fungi, Growth stage, Mycobiome, Pigs

## Abstract

**Background:**

The composition and relative abundances of intestinal microbiota are closely related to animal growth, development and health. This study provides a comprehensive analysis of the spatial distribution and temporal dynamics of intestinal fungi in pigs, with a focus on fungal alterations associated with diarrhea.

**Methods:**

Intestinal digesta from duodenum, jejunum, ileum, cecum, colon and feces of 8 finishing pigs (180 days old) were collected. Fecal samples were also collected from 18 pigs across different growth stages, including lactation (3 d), nursery (26 d, 35 d, 49 d), growing (120 d) and finishing (180 d). Additionally, feces were collected from 32 diarrheal and 32 healthy piglets at 28 days old. Fungal community profiling in these samples was performed using internal transcribed spacer (ITS) sequencing.

**Results:**

A total of 9,224 amplicon sequence variants (ASVs) were detected in all of 220 samples. Intestinal fungal diversity exhibited clear biogeographic patterns, with significantly lower richness and Shannon index in the ileum (*P* < 0.05) and significantly higher richness in the large intestine and feces (*P* < 0.05). The fungal community structure also varied significantly across intestinal segments, with *Kazachstania* dominating in the ileum and *Geotrichum* in the duodenum and jejunum. Across growth stages, fecal fungal diversity increased after weaning. PCoA results revealed that fungal structure exhibited significant temporal changes (*R* = 0.7313, *P* = 0.001), with the core fungal taxa dominated by *Diutina catenulata*, *Aspergillus restrictus* and *Tahromyces munnarensis*. In addition, by comparing the fungal community of piglets with and without diarrhea, the richness and Shannon index were significantly higher in the diarrheal piglets than those in healthy piglets (*P* < 0.05) with *Kazachstania*, *Diutina* and *Aspergillus* enriched in diarrheal piglets and *Geotrichum*, *Tahromyces* and *Piromyces* in healthy piglets.

**Conclusions:**

The intestinal fungal community in pigs shows distinct spatial variation, with greater diversity in the large intestine. Fungal composition shifts dynamically with age, particularly around the weaning transition. This study highlights specific fungal taxa associated with diarrhea caused by weaning stress, offering new insights into the interplay between gut fungi and pig health.

**Supplementary Information:**

The online version contains supplementary material available at 10.1186/s40104-025-01206-9.

## Background

The intestinal microbiota, including bacteria, archaea, fungi, protists, and viruses [1–3], play an important role in host health including energy metabolism [4–7], immunity and preventing pathogen invasion [8, 9]. Disruption of this complex microbial ecosystem could lead to host dysfunction and various diseases. Among these microbial components, fungi have emerged as key players that influence microbial community dynamics, modulate intestinal metabolite production, and interact with immune cells to maintain immune development and homeostasis [10]. Recent studies have highlighted association between intestinal fungi and health in both humans and animals [11–13].

Due to the unculturable nature of many intestinal fungi, characterizing the mycobiome using sequencing technologies has become increasingly important [14]. In humans, Ascomycota and Basidiomycota were the most abundant fungal phyla, showing a negative correlation and influencing the prevalence of inflammatory bowel disease [15]. Similar patterns have been observed in lambs, where these phyla remain dominant throughout growth, despite changes in fungal abundance driven by external environmental factors [16, 17]. Notably, specific fungi such as *Thermomyces* and *Saccharomyces* have been linked to body weight gain in mice by promoting lipid deposition [18] while *Wickerhamomyces*, *Meyerozyma*, and *Rhinocladiella* were closely associated with diarrhea in Baer’s pochards [19]. However, detailed understanding of the fungal composition and dynamics in the pig intestine remains sparse.

As an economically important species and a widely used biomedical model [6, 20–22], pigs provide an excellent system for studying intestinal microbiota. Previous longitudinal studies have revealed significant alterations in the microbiota during weaning, a critical transition from milk to solid feed [22]. This period of dietary and physiological changes often result in stress-induced diarrhea due to the immaturity of piglets' digestive and immune systems, leading to economic losses to the swine industry [23, 24]. Fungi might contribute to this phenomenon. For example, the role of *Candida tropicalis* in maintaining intestinal homeostasis has been noted but remains poorly understood [25].

Here, in this study, we aimed to characterize the fungal composition and spatial distribution across five intestinal segments (duodenum, jejunum, ileum, cecum, colon) and feces in pigs. A longitudinal study was also performed to explore the fungal succession throughout the growth stages. Furthermore, the differences in fungal communities between healthy and diarrheal weaned piglets were investigated to provide novel insights into the relationship between intestinal fungi and weaning stress-induced diarrhea.

## Materials and methods

### Experimental design and sample collection

All experiments were conducted in Zhejiang Province based on an experimental crowd of 1,400 Duroc × (Landrace × Yorkshire) crossbred newborn piglets. The define of growth periods as listed: Lactation period (d 0 to 21); Weaning period (d 21 to 28); Nursery (to d 49); Growing (to d 120); Finishing (to d 180). Throughout the experimental period, all pigs had ad libitum access to water and a commercial compound feed devoid of antibacterial additives and organic acids. Ingredients and nutrient composition of experimental diets in piglets were listed in Additional file 1: Tables S1, S2, and S3.

#### Exp. 1: Biogeography of the pig intestinal fungi

A total of 8 pigs with median of body weight were selected to sacrifice at 180 days old. The digesta from duodenum, jejunum, ileum, cecum, colon and fecal samples were collected in aseptic bags. The sampling methods were described as our previous experiments [26]. All samples were transferred to the laboratory on dry ice and frozen at −80 °C until internal transcribed spacer (ITS) sequencing.

#### Exp. 2: Succession of fecal fungi in pigs at different growth periods

A total of 18 healthy newborn piglets with initial body weight (1.67 ± 0.40 kg) were fed for 180 d in the present experiment. Fresh fecal samples were collected in the following stages: lactation (Day 3 [NB]), nursery (Day 26 [NP1], Day 35 [NP2], Day 49 [NP3]), growing (Day 120 [GF]), and finishing (Day 180 [FP]). The samples were transferred to the laboratory on dry ice and frozen at −80 °C until ITS sequencing. None of these pigs died during the whole experiment.

#### Exp. 3: Comparison of fungal composition between diarrheal and healthy piglets

Based on the experimental crowd of 1,400 newborn piglets, body weights and fecal diarrhea scores of piglets were recorded daily during the weaning period (d 21 to 28). Diarrhea scores were recorded as previously described: 0, solid; 1, semi-solid; 2, semi-liquid; 3, liquid [27]. Thirty-two piglets with continuous diarrhea scores ≥ 1 were selected as the diarrhea group [28], while 32 piglets with continuous diarrhea scores < 1 were categorized as the healthy group. On d 28, fecal samples from piglets in diarrheal and healthy groups were collected, and then transferred to the laboratory on dry ice and frozen at −80 °C until ITS sequencing.

### DNA extraction and sequencing

Total DNA of each sample was extracted using the QIAamp DNA stool mini kit (Qiagen, Hilden, Germany) following the manufacturer’s instructions. DNA quality and concentration of samples were measured with an ultraviolet spectrophotometer (Thermo Scientific, USA). The degradation of DNA was detected by agarose gel electrophoresis. High-quality DNA samples (A_260_/A_280_ ratio ≥ 1.80), with little degradation observed on agarose gels, were transported on the dry ice and sequenced for targeting the ITS2 region of the fungal rRNA gene. DNA was amplified using primer sequences ITS3 F (5'-GCATCGATGAAGAACGCAGC-3') and ITS4R (5'-TCCTCCGCTTATTGATATGC-3') [29, 30] on the Illumina platform. The sequencing data were subjected to quality control, denoising and deblurring processes on the QIIME 2 platform to obtain high-quality sequences [31]. The ASV (amplicon sequence variant) representative sequences were classified and subjected to statistical analysis using UCLUST software in combination with the UNITE reference database [32]. In this study, these sequences were denoted by fungal features, which were synonymous with ASV. Different feature sequences represented differences at the single nucleotide level [33].

### Bioinformatics and statistical analysis

The fungal diversity was analyzed based on the relative abundance of ASVs in each sample, including coverage, ACE, Chao1, Shannon’s index and Simpson’s index [34]. The principal coordinate analysis (PCoA) of the fungal communities based on Bray-Curtis dissimilarity distance matrices. Significance between community structure was evaluated by analysis of similarities (ANOSIM). Differential identification of the core fungi taxa was performed using linear discriminant analysis effect size (LEfSe) analysis at the feature level [35].

Statistical analysis of all data was performed with SPSS 25.0. Origin (version 2024) and GraphPad Prism software (version 9.5) were used to data visualization. The Kruskal-Wallis test was used to compare alpha-diversity of fungal communities in different intestinal digesta, and fecal samples at different stages. Student’s two-tailed unpaired *t*-test was used to compare the alpha-diversity of fungal communities between healthy and diarrheal groups. The results were expressed as the mean ± SEM (standard error of the mean). *P* < 0.05 was considered as significant difference.

## Results

### Description of sequencing data

The dynamics of the intestinal fungal community in pigs were characterized by collecting 220 samples. A total of 48 digesta and fecal samples were collected from various intestinal sections (duodenum, jejunum, ileum, cecum, and colon) at 180 days old. Additionally, 108 feces were sampled from 18 pigs at different stages of development (d 3, 26, 35, 49, 120, and 180). Furthermore, 64 fecal samples were obtained from 32 healthy piglets and 32 diarrheal piglets. A total of 9,224 ASVs were measured, generating 8,166,180 high-quality reads with an average of 37,119 reads per sample.

**Fig. 1:**
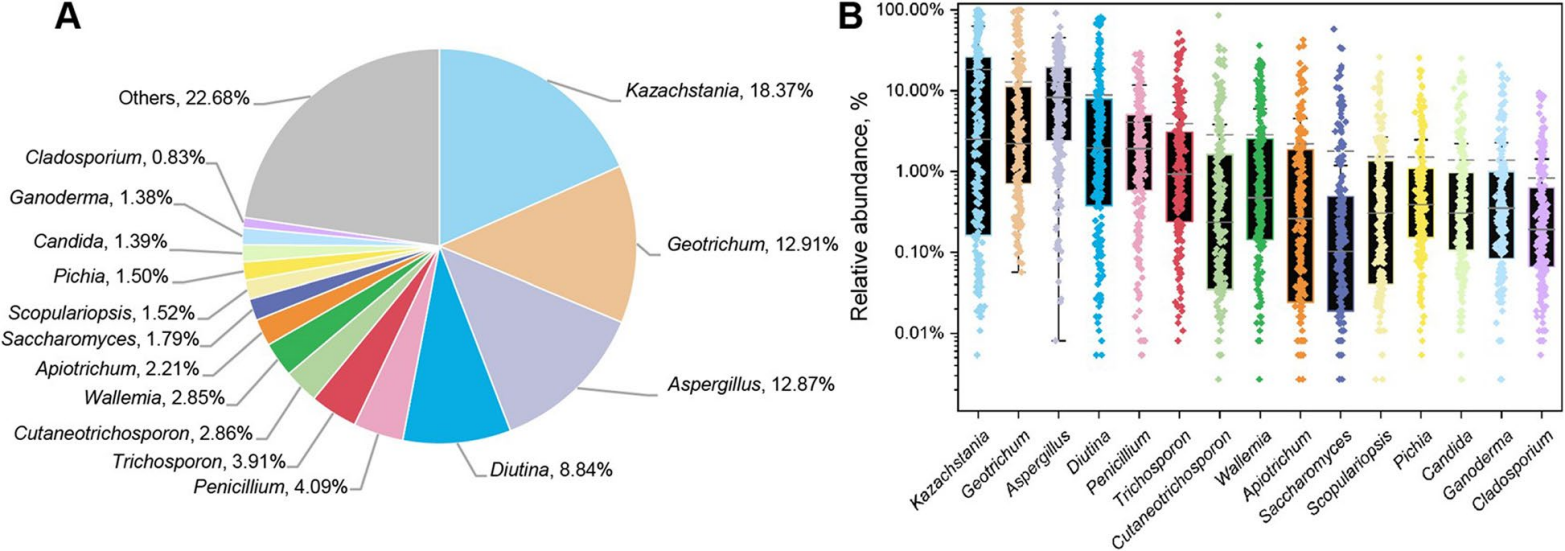
The proportion of each genera in all sequences combined (**A**) and the abundance distribution of top 15 abundant fungal genera (**B**)

We further analyzed the 15 most abundant fungal genera, accounting for 77.32% of the total sequences (Fig. 1A). The abundant genera were mainly classified as phyla Ascomycota and Basidiomycota (Fig. 1B and Additional file 2: Fig. S1). In addition, the relative abundance of fungal genera varied considerably across samples.

### Biogeography of the pig intestinal fungi

**Fig. 2:**
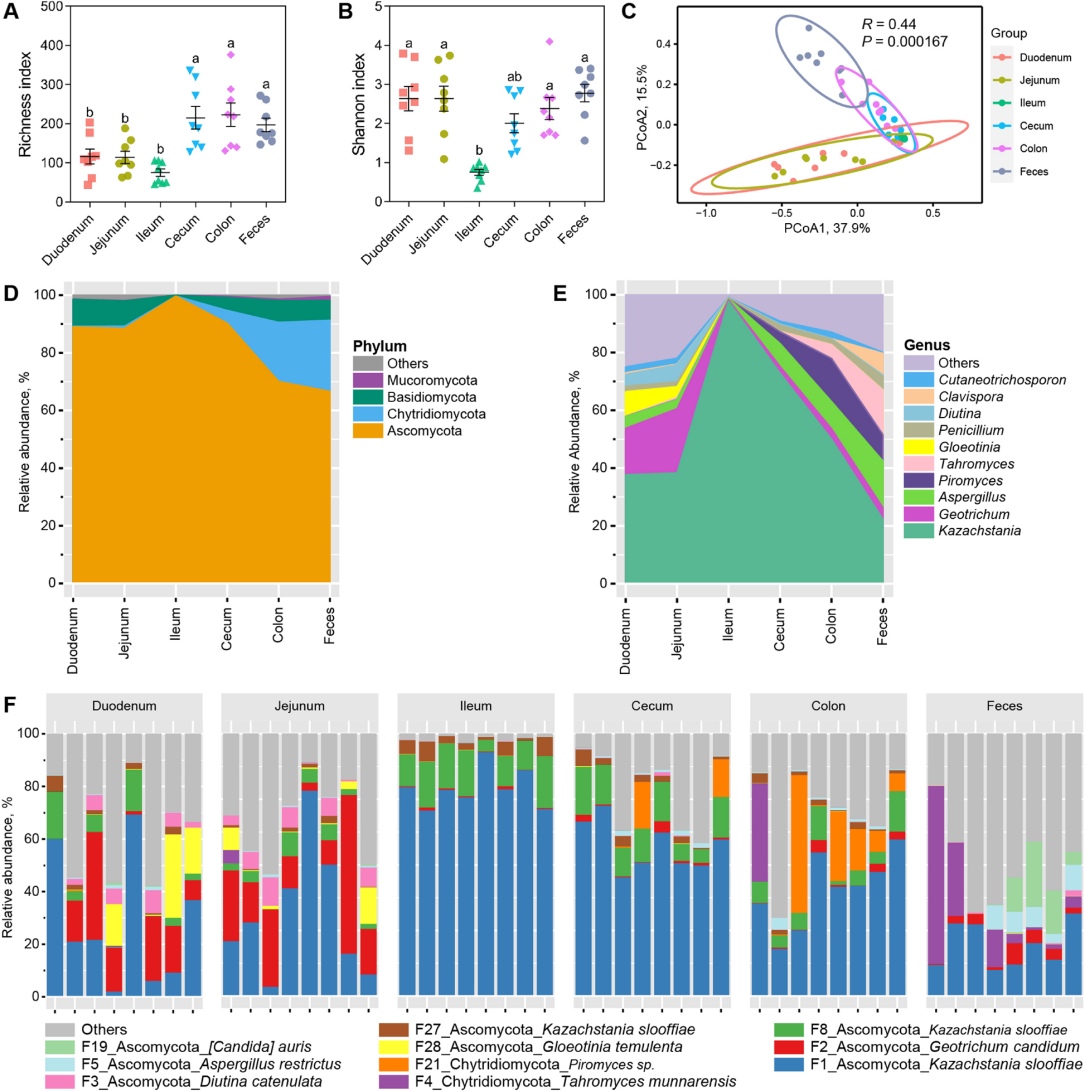
Diversity and biogeography of intestinal and fecal fungi. The richness (**A**) and Shannon index (**B**) show the α-diversity of the intestinal and fecal fungi. Significance is determined by Kruskal-Wallis test and presented as mean ± SEM. PCoA (**C**) of the intestinal and fecal fungi. Mean relative abundances of top 10 abundant phyla (**D**) and genera (**E**) in intestinal digesta and feces. The relative abundance of top 10 abundant features (**F**) is indicated by individual samples

Alpha-diversity of intestinal and fecal fungi was analyzed with richness and Shannon indices. The richness of the cecal, colonic, and fecal fungi was significantly higher than that of the duodenum, jejunum and ileum (*P* < 0.05, Fig. 2A). The Shannon index was significantly lower in the ileum than other segments (*P* < 0.05, Fig. 2B). It indicated that the diversity of fungi increased from proximal to distal intestinal segments, and obviously decreased in the ileum. The PCoA showed significant clustering of different intestines (Fig. 2C), indicating significant differences in the fungal structure between the small intestine (duodenum, jejunum and ileum) and large intestine (cecum and colon) of pigs (*R* = 0.44, *P* < 0.001).

At the phylum level, Ascomycota, Basidiomycota, and Chytridiomycota dominated intestinal digesta and feces (Fig. 2D), accounting for over 98% relative abundance. Ascomycota enriched in small intestine and decreased in large intestine, while Basidiomycota and Chytridiomycota showed a reverse change pattern. At the genus level, a total of 438 genera were identified in intestinal digesta and feces of pigs. The top 10 abundant genera accounted for over 75% relative abundance (Fig. 2E). *Kazachstania* was the dominant genera, accounting for over 20% relative abundance, followed by *Geotrichum* and *Aspergillus*. In addition, the relative abundance of *Kazachstania* increased through the small intestine, hitting its peak in the ileum, and then decreased through the large intestine. *Geotrichum* was the dominant fungal genera in the duodenum and jejunum, and *Piromyces* was most abundant in the colon. In addition, the dominant fungal genera were changed to *Aspergillus* and *Tahromyces* in the feces (Fig. 2F).

**Fig. 3:**
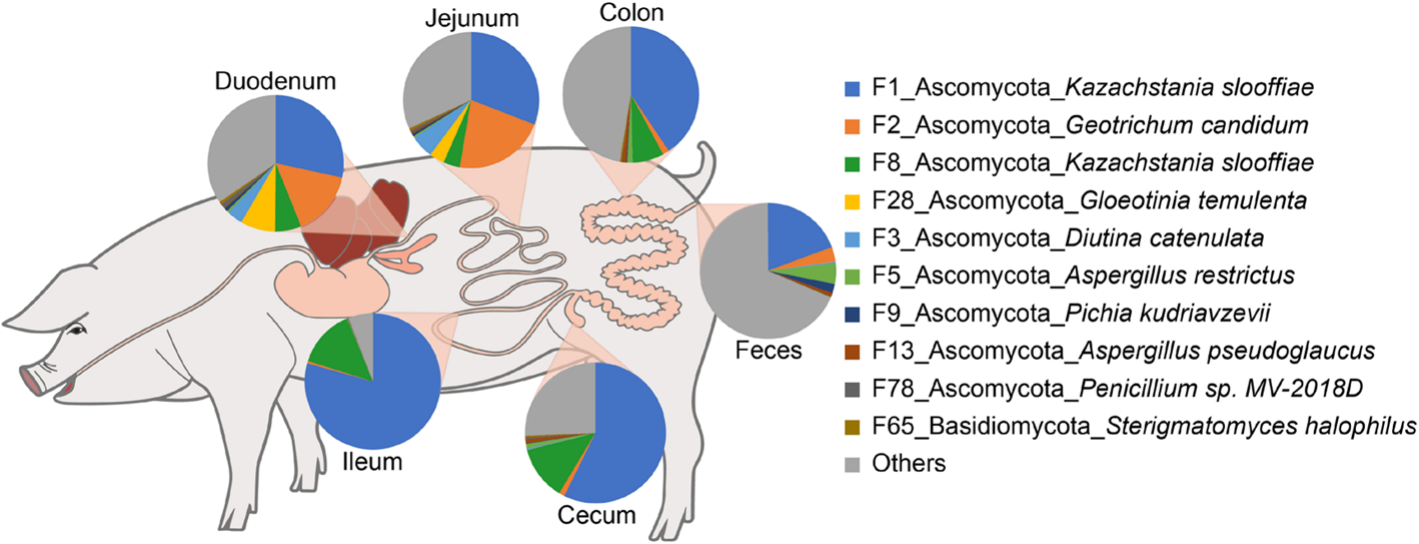
Biogeography of the core fungal taxa in 180-day-old pigs. Relative abundances of the 10 most abundant features are shown

Since fungal composition varied widely among individuals, an attempt was made to compare differences among different intestinal digesta and feces by identifying core mycobiome. The core fungal taxa was defined as the fungal ASVs present in over 80% of sequenced samples [30]. A total of 10 abundant fungal features were identified as core fungal taxa in the intestine (Fig. 3). The core fungal taxa were dominated by *Kazachstania slooffiae* throughout the gastrointestinal tract. The core features belonged primarily to phylum Ascomycota.

### Succession of the fecal fungi in pigs

**Fig. 4:**
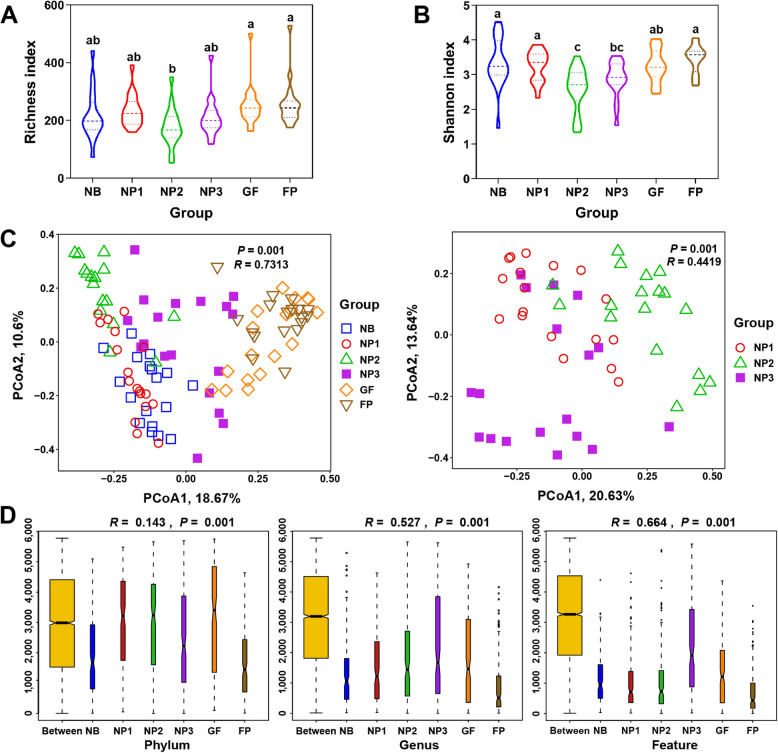
Characterization of longitudinal changes in pig intestinal fungi. Richness index (**A**), Shannon index (**B**), PCoA (**C**) and ANOSIM of intestinal fungi at the phylum, genus and feature level (**D**). The difference is determined by Kruskal-Wallis test. *NB* lactation piglets (3 d), *NP1* Nursery piglets (26 d), *NP2* Nursery piglets (35 d), *NP3* Nursery piglets (49 d), *GF* Growing pigs (120 d), *FP* Finishing pigs (180 d). ^a–c^Different letters indicate significant differences (*P* < 0.05)

To understand the succession of fecal fungi in pigs, we first analyzed the α-diversity of fecal fungi with richness and Shannon indices at different growth stages. The richness index of NP2 was significantly lower than that of GF and FP (*P* < 0.05), while there was no significant change between GF and FP (Fig. 4A). The Shannon index of NP2 was significantly lower than that of NB, NP1, GF and FP (*P* < 0.05). Moreover, there was no difference among the stages of NB, NP1, GF and FP (Fig. 4B). In general, the richness and Shannon indices of fecal fungi were at a lower level before weaning, followed by a gradual increase. PCoA results revealed that fungal structure exhibited significantly temporal changes (*R* = 0.7313, *P* = 0.001, Fig. 4C). PC1 axis also displayed distinct longitudinal patterns of fungi throughout the nursery period (*R* = 0.4419, *P* = 0.001). In addition, the ANOSIM analysis showed that the fungal communities of NB, NP1, NP2, NP3, GF, and FP were all changed at the phylum, genus and feature level (Fig. 4D).

### The composition of fecal fungi in pigs at different growth stages

**Fig. 5:**
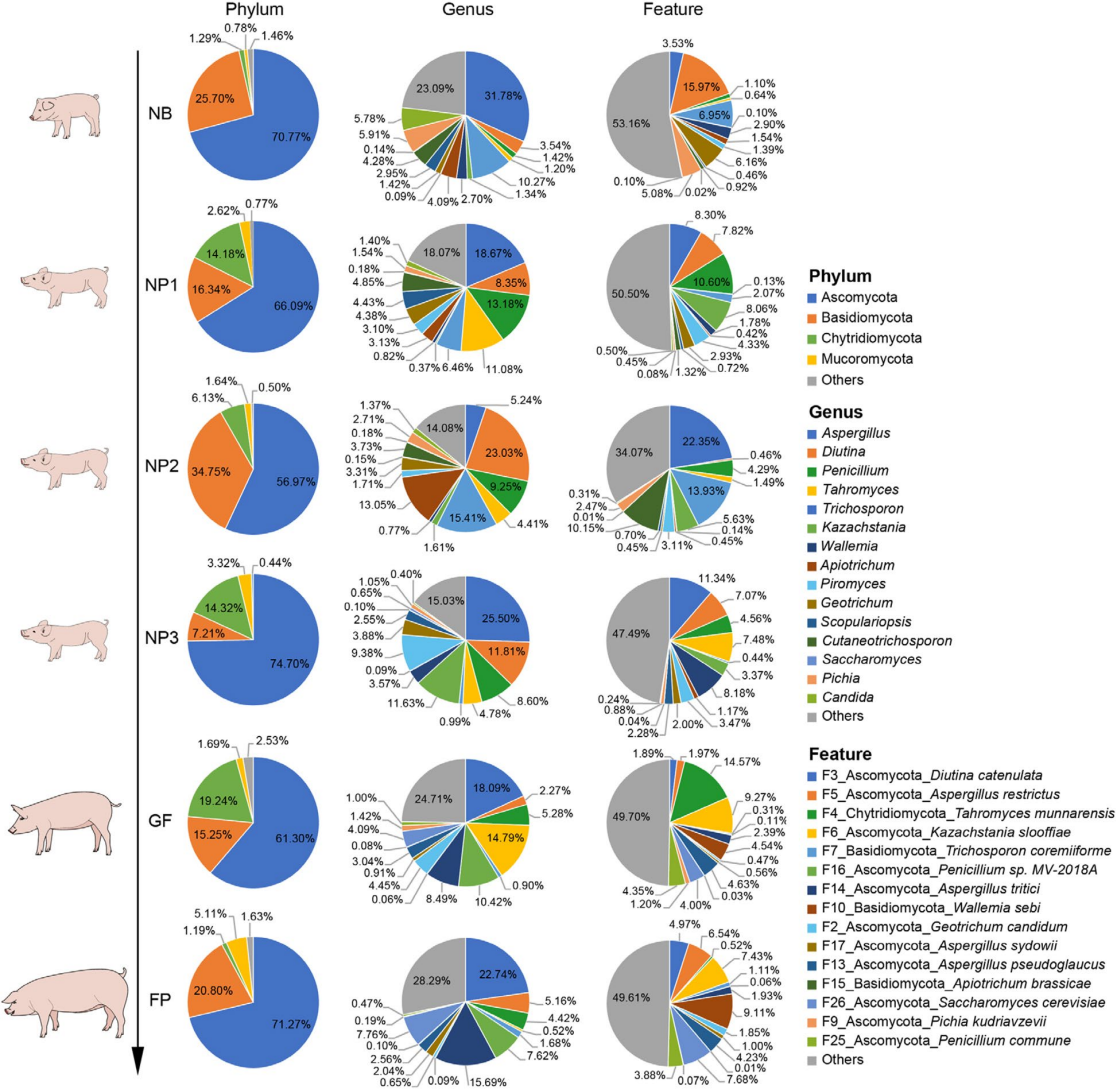
The composition of fecal fungi in different stages of pigs is shown at phylum, genus and feature level. *NB* lactation piglets (3 d), *NP1* Nursery piglets (26 d), *NP2* Nursery piglets (35 d), *NP3* Nursery piglets (49 d), *GF* Growing pigs (120 d), *FP* Finishing pigs (180 d)

At the phylum level, the dominant phyla were Ascomycota, Basidiomycota and Chytridiomycota (Fig. 5 and Additional file 2: Fig. S2). Ascomycota was the most abundant phylum, ranging from 56.97% to 74.70%. Basidiomycota enriched in NP2 period (34.75%) and decreased in NP3 period (7.21%). Chytridiomycota was abundant in NP1, NP2, NP3 and GF periods, while decreased in NB and FP periods. At the genus level, the dominant genera were not consistent. The top 10 abundant genera were *Aspergillus*, *Diutina*, *Penicillium*, *Tahromyces*, *Trichosporon*, *Kazachstania*, *Wallemia*, *Apiotrichum*, *Piromyces* and *Geotrichum*. The highest abundance of *Aspergillus* was found in NB (31.78%) period and the lowest in NP2 (5.24%) period. *Diutina* and *Trichosporon* dominated in NP2 period, accounting for 23.03% and 15.41%, respectively. At the feature level, the three most abundant features were *Diutina catenulata*, *Aspergillus restrictus* and *Tahromyces munnarensis*. *Diutina catenulata* was the most abundant feature in NP2 and NP3 periods, accounting for 22.35% and 11.34%, respectively. *Aspergillus restrictus* was the most abundant feature in NB period, accounting for 15.97%. *Tahromyces munnarensis* was the most abundant feature in NP1 and GF periods, accounting for 10.60% and 14.57%, respectively.

**Fig. 6:**
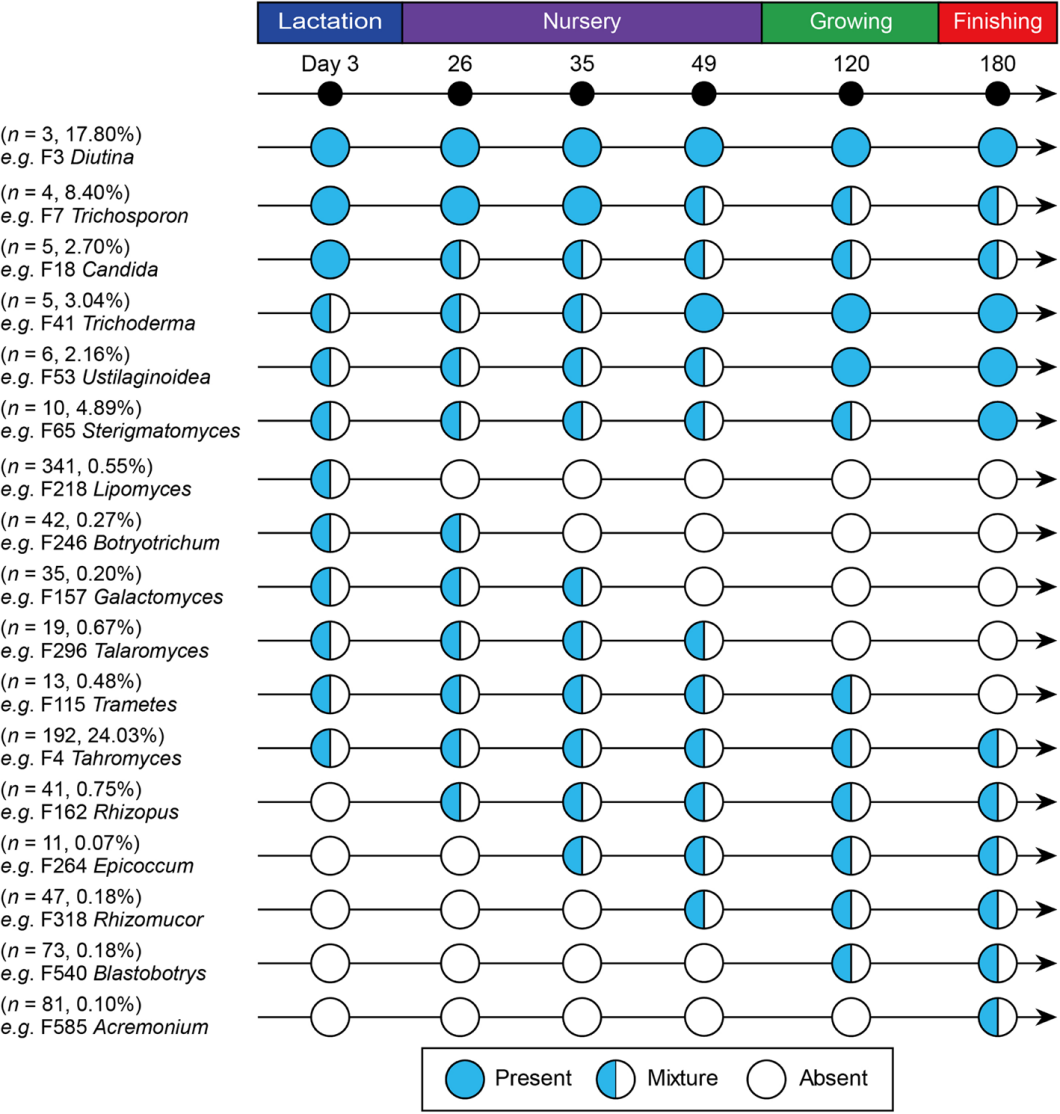
Longitudinal occurrence patterns of fecal fungal members in pigs. The occurrence patterns of the top 2,000 features according to mean relative abundance at each stage are summarized. Blue circles indicate the presence of a fungal taxa, while white circles indicate absence. The mixed color circle is a transition between presence and absence at a certain stage

During the whole lifetime of pigs, three features were consistently observed, which were defined as core fungal taxa, including *Diutina catenulata*, *Aspergillus restrictus* and *Tahromyces munnarensis* (Fig. 6). In addition, 30 features consistently exist at a particular stage. For example, *Candida tropicalis* and *Sterigmatomyces halophilus* enriched in the lactation and finishing periods, respectively, and disappeared at other periods (Additional file 2: Fig. S3–S5). A total of 895 features were known as passengers, appearing sporadically and disappearing completely at some time.

### Comparison of fungal composition between diarrheal and healthy piglets

**Fig. 7:**
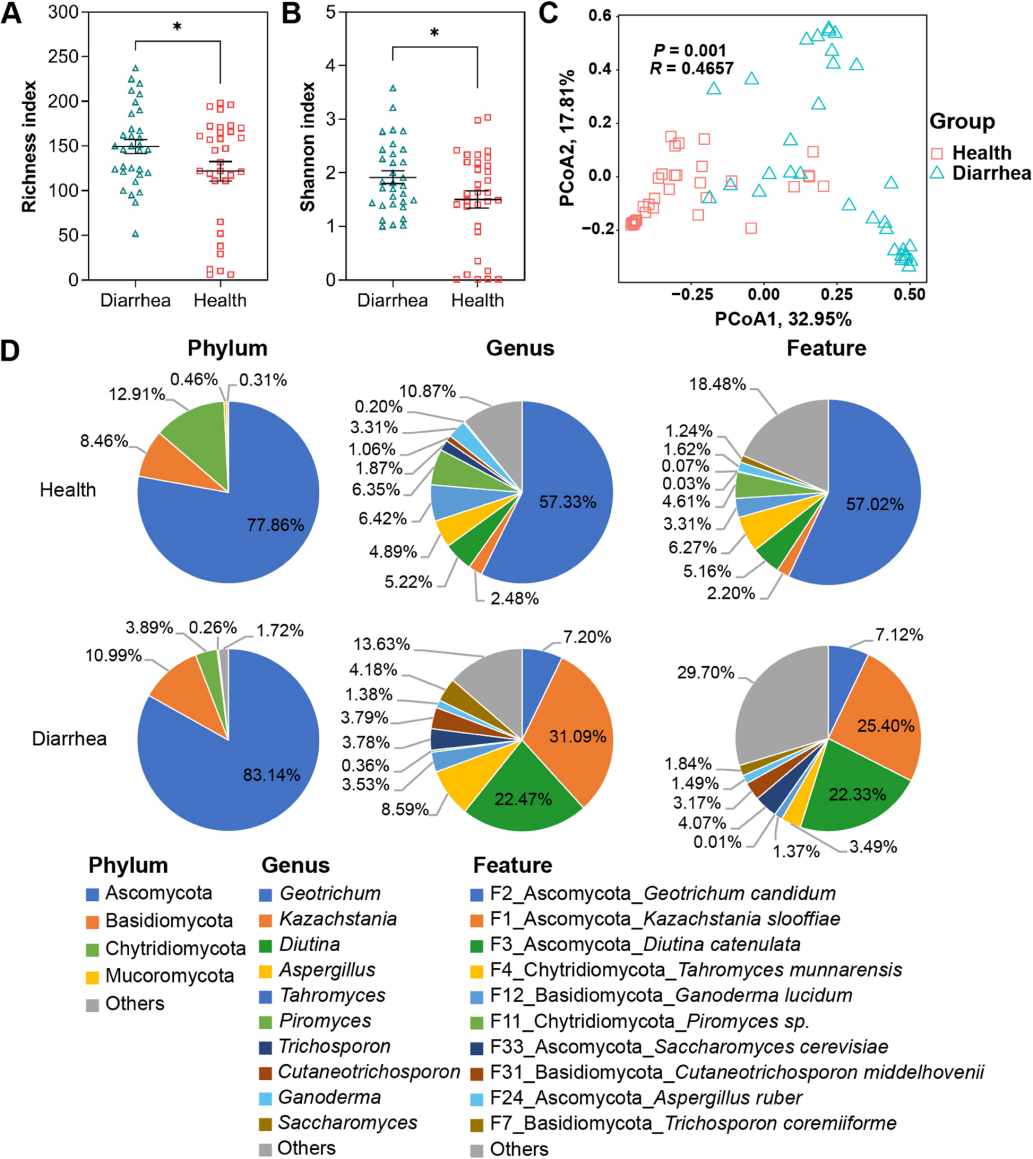
Comparative analysis of fecal fungal diversity between healthy and diarrheal piglets. Richness index (**A**), Shannon Index (**B**), PCoA (**C**) and composition of fecal fungi at the phylum, genus and feature level (**D**). Significance is determined by independent-sample *t*-test and presented as mean ± SEM, ^*^P < 0.05

The richness and Shannon indices of diarrheal piglets were significantly higher than those of healthy piglets (Fig. 7A and B; *P* < 0.05). The PCoA revealed distinct clustering of fecal fungi communities between healthy and diarrheal piglets (Fig. 7C), indicating notable differences in fungal composition. At phylum level, Chytridiomycota was enriched in healthy piglets, while Ascomycota dominated in diarrheal piglets (Fig. 7D). At genus level, *Geotrichum* was the dominant fungal genus in healthy piglets, followed by *Tahromyces* and *Piromyces*. *Kazachstania*, *Diutina* and *Aspergillus* were predominant in diarrheal piglets. At the species level, *Geotrichum candidum* and *Tahromyces munnarensis* were the most abundant in healthy piglets, whereas *Kazachstania slooffiae* and *Diutina catenulata* were enriched in diarrheal piglets.

**Fig. 8:**
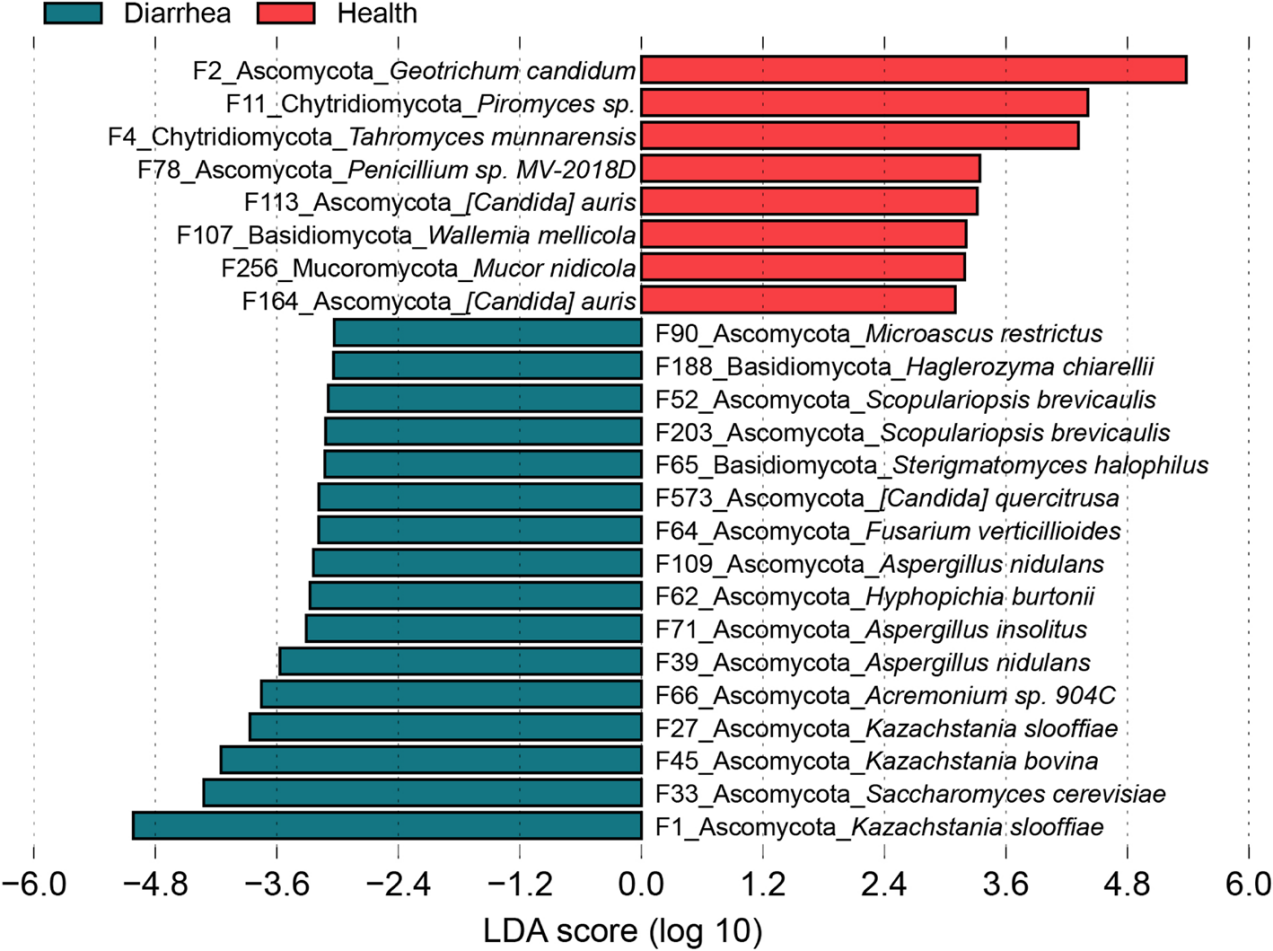
LDA score shows 24 fungal taxa identified in healthy and diarrheal piglets. The top 500 abundant fungal features are used for LEfSe analysis (LDA > 3.0)

The LEfSe analysis showed that *Geotrichum candidum*, *Piromyces *sp. and *Tahromyces munnarensis* mainly enriched in healthy piglets, while *Kazachstania slooffiae*, *Saccharomyces cerevisiae*, *Kazachstania bovina*, *Kazachstania slooffiae* and *Acremonium *sp. 904 C mainly enriched in diarrheal piglets (Fig. 8 and Additional file 2: Fig. S6). In addition, abundant pathogenic fungi enriched in diarrheal piglets, such as *Aspergillus nidulans*, *Aspergillus insolitus*, *Hyphopichia burtonii*, *Aspergillus nidulans* and *Fusarium verticillioides*.

## Discussion

The intestinal microbiota affects host health, metabolism and reproduction [36, 37]. Fungi were considered as important members of the intestinal microbiota, playing a crucial role in the host’s health [10]. The present study offered a comprehensive overview of pig intestinal fungi by analyzing digesta and feces collected from various intestinal segments, developmental stages, and physiological states. Building on this detailed mapping of the pig intestinal mycobiome, we further investigated the fungal distribution in both diarrheal and healthy piglets to uncover key differences associated with health status.

The fungal communities in different intestinal segments exhibited clear biogeographic pattern. Previous study found that the fungal diversity in pig intestine was decreasing and then increasing [38]. In our study, there were differences in the composition of fungal community among distinct intestinal digesta. As expected, fungi were more abundant in the large intestine than in the small intestine. The fungal diversity decreased from the proximal intestine to the ileum and then increased gradually to the distal intestine. The core fungal taxa were dominated by *Kazachstania slooffiae* throughout the gastrointestinal tract, which was consistent with the previous study [39]. Several studies indicated that *Kazachstania* was a characteristic fungus of the pig intestine [40] and regulated intestinal metabolism by promoting intestinal glycolysis and increasing adenosine triphosphate (ATP) production [41]. *Kazachstania slooffiae* played a probiotic role in the digestive system of pigs and was a good source of protein for animals [42]. *Geotrichum candidum* was considered to have an anti-inflammatory effect on epithelial cells and reduced interleukin-8 (IL-8) production, which had been demonstrated to benefit growth and immunity in animals [43, 44].

Weaning might be one of the key factors that could not be ignored for the alteration of fungal community in pigs during the growth stages. The PCoA showed that fungal composition changed dynamically throughout the production cycle. Interestingly, the fungal community structure was completely changed after weaning. *Candida tropicalis* enriched in piglets during the lactation period. The occurrence of opportunistic pathogenic species such as *Candida tropicalis* posed a potential threat to young animals whose immune systems were not yet fully developed [45]. In the finishing period, *Sterigmatomyces halophilus* had been found to modulate immunity in pigs [46]. The fungal diversity gradually increased after weaning, which was consistent with the results of Summers et al. [47]. In addition, *Diutina catenulate* had the potential to induce infections in immunocompromised animals [48]. During the transition from the lactation stage to the nursery stage, there was a significant increase in the relative abundance of *Diutina catenulate*. Moreover, from the nursery stage to the finishing stage, the relative abundances of *Kazachstania slooffiae* and *Saccharomyces cerevisiae* rose significantly. Yin et al. [16] found that aging increased the abundance of *Saccharomyces cerevisiae*, which may have played an important role in fungal community maturation and helped animals digest carbohydrate-rich diets. Previous study found that *Saccharomyces cerevisiae* not only modulates the balance of the intestinal microbiota and inflammatory responses [49], but also enhances the growth performance and alleviates diarrhea in weaned piglets [50]. *Saccharomyces cerevisiae* was present in < 1% relative abundance or absent in weaned healthy piglets [51], which was consistent with our results. In the present study, *Saccharomyces cerevisiae* enriched in diarrheal piglets. Holanda et al. also found it in feces with diarrhea [52]. Weaning caused strong stress on physiologically immature piglets, which potentially resulted in an increased risk of fungal infections [53]. *Saccharomyces cerevisiae* might have a particular effect under different host status. In detail, it generally promotes the growth performance [49, 50], while increasing the occurrence of diarrhea under weaning stress in pigs [52, 54, 55].

In the present study, the relative abundance of *Geotrichum candidum*, *Piromyces *sp*.* and *Tahromyces munnarensis* was significantly higher in healthy piglets compared to diarrheal piglets. *Geotrichum candidum* was classified as a yeast species that exhibited a pronounced inhibitory effect on fungal contaminants, competed with undesirable microorganisms for substrates and space, and was ubiquitous in numerous habitats such as forage, soil, plants, humans and other mammals [56]. Yeast could increase the concentration of anti-inflammatory factors and was related to the polysaccharide metabolism in diets containing mannan and glucan [57], which exerted a positive influence on intestinal health [58]. Functional lytic polysaccharide monooxygenases encoded by *Geotrichum candidum* were active on cellulose and xyloglucan [59], which was beneficial for growth performance and feed digestion [60–62]. Both *Piromyces* and *Tahromyces* were able to utilize cellulose, xylose and glucose and potentially played a pivotal role in the degradation process of plant cell walls [63, 64]. Mycotoxins, mainly generated by *Aspergillus*, *Fusarium* and *Penicillium*, were secondary metabolites produced by filamentous fungi, that would be potentially toxic to pigs and might induce diarrhea [65]. The present study also found that the relative abundances of *Aspergillus nidulans*, *Aspergillus insolitus*, *Aspergillus nidulans* and *Fusarium verticillioides* were significantly increased in diarrheal piglets compared to healthy piglets. Aflatoxin and fumonisin were carcinogenic mycotoxins produced by *Aspergillus* and *Fusarium*, respectively, which caused intestinal disturbances and led to diarrhea [66–68]. Mycotoxins exhibited a synergistic toxic effect, damaging the growth performance of pigs [68]. A study showed that *Hyphopichia burtonii* caused fungal peritonitis in patients [69, 70]. In this research, *Hyphopichia burtonii* was classified as one of the fungal biomarkers associated with diarrheal piglets with the potential to cause diarrhea in piglets.

## Conclusions

In conclusion, this study provides valuable insights into the spatial distribution and temporal dynamics of intestinal fungi in pigs. Distinct variations in fungal diversity across different intestinal segments were investigated, with a higher richness and Shannon indices observed in the large intestine compared to the small intestine. The fungal community shifted significantly during the growth stages, with core fungal taxa dominated by *Kazachstania*, *Geotrichum*, and *Aspergillus* playing key roles at different developmental points. Notably, the fungal structure in diarrheal piglets showed distinct differences compared to healthy piglets, with *Kazachstania* and *Diutina* being more abundant in the diarrheal group, while *Geotrichum* and *Tahromyces* dominated in healthy piglets. These findings suggest that specific fungal taxa may be associated with diarrhea in piglets, providing potential targets for future research on gut health management and disease prevention strategies in swine production.

## Supplementary Information

Additional file 1: Table S1 Ingredients and nutrient composition of experimental diets for sows during gestation and lactation. Table S2 Ingredients and nutrient composition of experimental diets in piglets. Table S3 Ingredients and nutrient composition of experimental diets in the growing pigs and finishing pigs.

Additional file 2: Fig. S1 Phylogenetic relationships of the top 100 most abundant fungi. Fig. S2 Top 30 fungi in different stages of pigs. Fig. S3 Heatmap of stage-related fungi. Fig. S4 Network analysis of interactions at different growth stages. Spearman was used to calculate the top 600 fungi. Fig. S5 Regression-based random forest algorithm was used to select the top 50 growth-related fungi from the top 600 fungi. Fig. S6 Heatmap shows 99 fungi identified by LEfSe in healthy and diarrheal piglets. The top 500 relative abundances were used for LEfSe analysis.

## Data Availability

The datasets supporting the conclusions of this article are available in the NCBI Sequence Read Archive (SRA) repository under accession number PRJNA1157718.

## Abbreviations

ANOSIM: Analysis of similarities
ASV: Amplicon sequence variant
ATP: Adenosine triphosphate
IL-8: Interleukin-8
ITS: Internal transcribed spacer
LEfSe: Linear discriminant analysis effect size
PCoA: Principal coordinate analysis
