# Supplementary material for: Intestinal fungi biogeography, succession and its association with diarrhea in pigs
Source: J Anim Sci Biotechnol. 2025 Jun 4;16:80. doi: 10.1186/s40104-025-01206-9 (PMC12135602; doi:10.1186/s40104-025-01206-9)
Supplement: Supplementary file 1 — Additional file 1: Table S1 Ingredients and nutrient composition of experimental diets for sows during gestation and lactation. Table S2 Ingredients and nutrient composition of experimental diets in piglets. Table S3 Ingredients and nutrient composition of experimental diets in the growing pigs and finishing pigs. [file 40104_2025_1206_MOESM1_ESM.docx]

**Table S1** Ingredients and nutrient composition of experimental diets for sows during gestation and lactation (%, as-fed basis).

| Items | Gestation | Lactation |
| --- | --- | --- |
| Ingredients |  |  |
| Corn | 63.17 | 58.81 |
| Soybean meal | 16.72 | 26.50 |
| Wheat bran | 14.00 | 8.71 |
| Soybean oil | 2.50 | 2.50 |
| Calcium hydrophosphate | 0.81 | 1.45 |
| Limestone | 1.17 | 1.05 |
| Salt | 0.50 | 0.50 |
| L-Lysine | 0.24 | 0.08 |
| Threonine | 0.05 | - |
| Tryptophan | 0.01 | - |
| Premix^1^ | 0.50 | 0.50 |
| Total | 100 | 100 |
| Nutrients levels^2^ |  |  |
| Digestive energy, MJ/kg ^2^ | 3269 | 3332 |
| Dry matter | 88.73 | 88.04 |
| Crude protein | 15.12 | 18.11 |
| Calcium | 0.68 | 0.80 |
| Total Phosphorus | 0.58 | 0.68 |
| Available phosphorus | 0.29 | 0.34 |

^1^Premix provided the following per kilogram of feed: vitamin A, 12,000 IU; vitamin E, 24 IU; vitamin K3, 2.0 mg; thiamine, 2.0 mg; riboflavin, 6.0 mg; pyridoxine, 4.0 mg; vitamin B12, 24 μg; niacin, 30 mg; pantothenic acid, 20 mg; folic acid, 3.6 mg; biotin, 0.4 mg; choline chloride, 0.4 mg; iron, 96 mg; copper, 8.0 mg; zinc, 120 mg; manganese, 40 mg; iodine, 0.56 mg; selenium, 0.4 mg.

^2^Calculated values.

**Table S2** Ingredients and nutrient composition of experimental diets in the nursery period (%, as-fed basis).

| Items | Nursery [NP1] | Nursery [NP2 and NP3] |
| --- | --- | --- |
| Ingredients |  |  |
| Corn | 37.53 | 60.79 |
| Soybean meal | 18.00 | 18.00 |
| Extruded corn | 20.00 | 8.00 |
| Protein whey powder | 8.00 | 4.00 |
| Fish meal | 5.00 | 2.00 |
| Soy protein concentrate | 3.00 | 2.00 |
| Soybean oil | 0.90 | 2.07 |
| Glucose | 2.50 | - |
| Sucrose | 2.00 | - |
| Dicalcium phosphate dihydrate | 0.53 | 0.80 |
| Limestone | 0.70 | 0.90 |
| L-Lysine | 0.57 | 0.40 |
| Methionine | - | 0.09 |
| Threonine | - | 0.13 |
| Tryptophan | - | 0.02 |
| Salt | 0.19 | 0.30 |
| Compound amino acids | 0.58 | - |
| Premix^1^ | 0.50 | 0.50 |
| Total | 100 | 100 |
| Nutrients levels^2^ |  |  |
| Digestive energy, MJ/kg | 14.35 | 14.70 |
| Crude protein | 20.21 | 18.98 |
| Calcium | 0.70 | 0.70 |
| Phosphorus | 0.61 | 0.54 |

^1^Premix provided the following per kilogram of feed:

Nursery [NP1]: vitamin A, 10,000 IU; vitamin D3, 2,300 IU; vitamin E, 25 IU; vitamin K3, 2.4 mg; thiamine, 2.0 mg; riboflavin, 4.0 mg; pyridoxine, 3.0 mg; vitamin B12, 12 μg; niacin, 30 mg; pantothenic acid, 13 mg; folic acid, 1.0 mg; biotin, 50 μg; iron, 9.0 mg; copper, 110 mg; zinc, 90 mg; manganese, 20 mg; iodine, 0.35 mg; selenium, 0.3 mg.

Nursery [NP2 and NP3]: vitamin A, 12,000 IU; vitamin D3, 2,500 IU; vitamin E, 30 IU; vitamin K3, 3.0 mg; vitamin B12, 12 μg; niacin, 30 mg; pantothenic acid, 10 mg; choline chloride, 400 mg; biotin, 0.4 mg; iron, 90 mg; copper, 100 mg; zinc, 100 mg; manganese, 40 mg; iodine, 0.35 mg; selenium, 0.3 mg.

^2^Calculated values.

**Table S3** Ingredients and nutrient composition of experimental diets in the growing and finishing period (%, as-fed basis).

| Items | Growing | Finishing |
| --- | --- | --- |
| Ingredients |  |  |
| Corn | 71.34 | 76.76 |
| Soybean meal | 20.21 | 8.40 |
| Wheat bran | 2.70 | 9.50 |
| Soybean oil | 2.11 | 2.11 |
| Dicalcium phosphate dihydrate | 0.68 | 0.60 |
| Limestone | 1.41 | 0.94 |
| L-Lysine | 0.40 | 0.45 |
| Methionine | 0.04 | 0.04 |
| Threonine | 0.07 | 0.15 |
| Tryptophan | 0.02 | 0.03 |
| Valine | 0.09 | 0.09 |
| Salt | 0.35 | 0.35 |
| Choline | 0.08 | 0.08 |
| Premix^1^ | 0.50 | 0.50 |
| Total | 100 | 100 |
| Nutrients levels^2^ |  |  |
| Digestive energy, MJ/kg | 14.20 | 14.16 |
| Crude protein | 15.88 | 12.18 |
| Calcium | 0.75 | 0.54 |
| Phosphorus | 0.50 | 0.50 |

^1^Premix provided the following per kilogram of feed: vitamin A, 12,000 IU; vitamin D, 3,000 IU; vitamin E, 30 IU; vitamin K, 2.5 mg; vitamin B12, 20 μg; riboflavin, 4.0 mg; niacin, 40 mg; pantothenic acid, 12.5 mg; choline chloride, 400 mg; folic acid, 0.7 mg; thiamine, 2.5 mg; pyridoxine, 3.0 mg; biotin, 70 μg; iron, 77 mg; copper, 20 mg; zinc, 69 mg; manganese, 14 mg; iodine, 0.43 mg; selenium, 0.43 mg.

^2^Calculated values.
