# Supplementary material for: Intestinal fungi biogeography, succession and its association with diarrhea in pigs
Source: J Anim Sci Biotechnol. 2025 Jun 4;16:80. doi: 10.1186/s40104-025-01206-9 (PMC12135602; doi:10.1186/s40104-025-01206-9)
Supplement: Supplementary file 2 — Additional file 2: Fig. S1 Phylogenetic relationships of the top 100 most abundant fungi. Fig. S2 Top 30 fungi in different stages of pigs. Fig. S3 Heatmap of stage-related fungi. Fig. S4 Network analysis of interactions at different growth stages. Spearman was used to calculate the top 600 fungi. Fig. S5 Regression-based random forest algorithm was used to select the top 50 growth-related fungi from the top 600 fungi. Fig. S6 Heatmap shows 99 fungi identified by LEfSe in healthy and diarrheal piglets. The top 500 relative abundances were used for LEfSe analysis. [file 40104_2025_1206_MOESM2_ESM.docx]

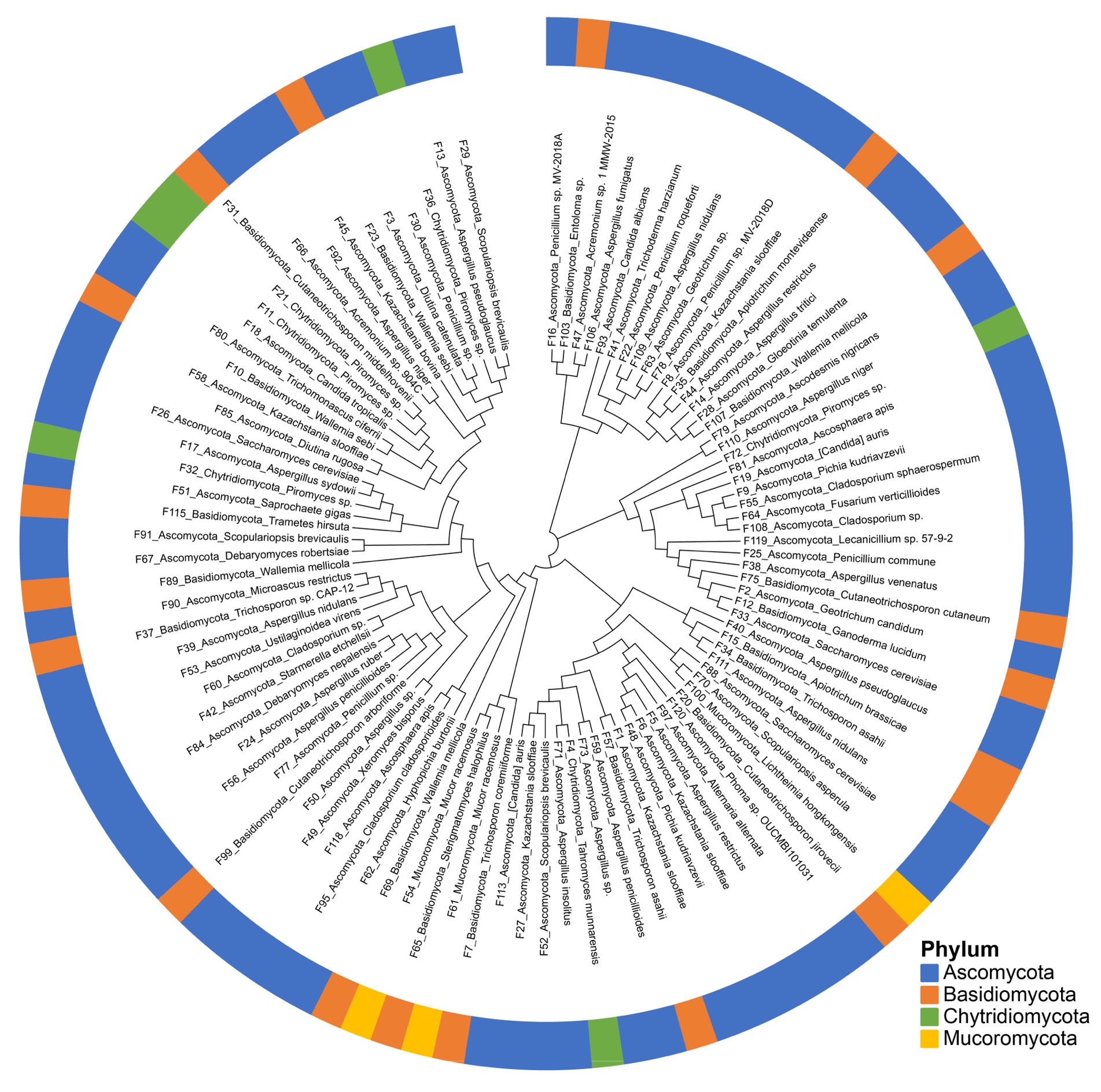


**Fig. S1** Phylogenetic relationships of the top 100 most abundant fungi.


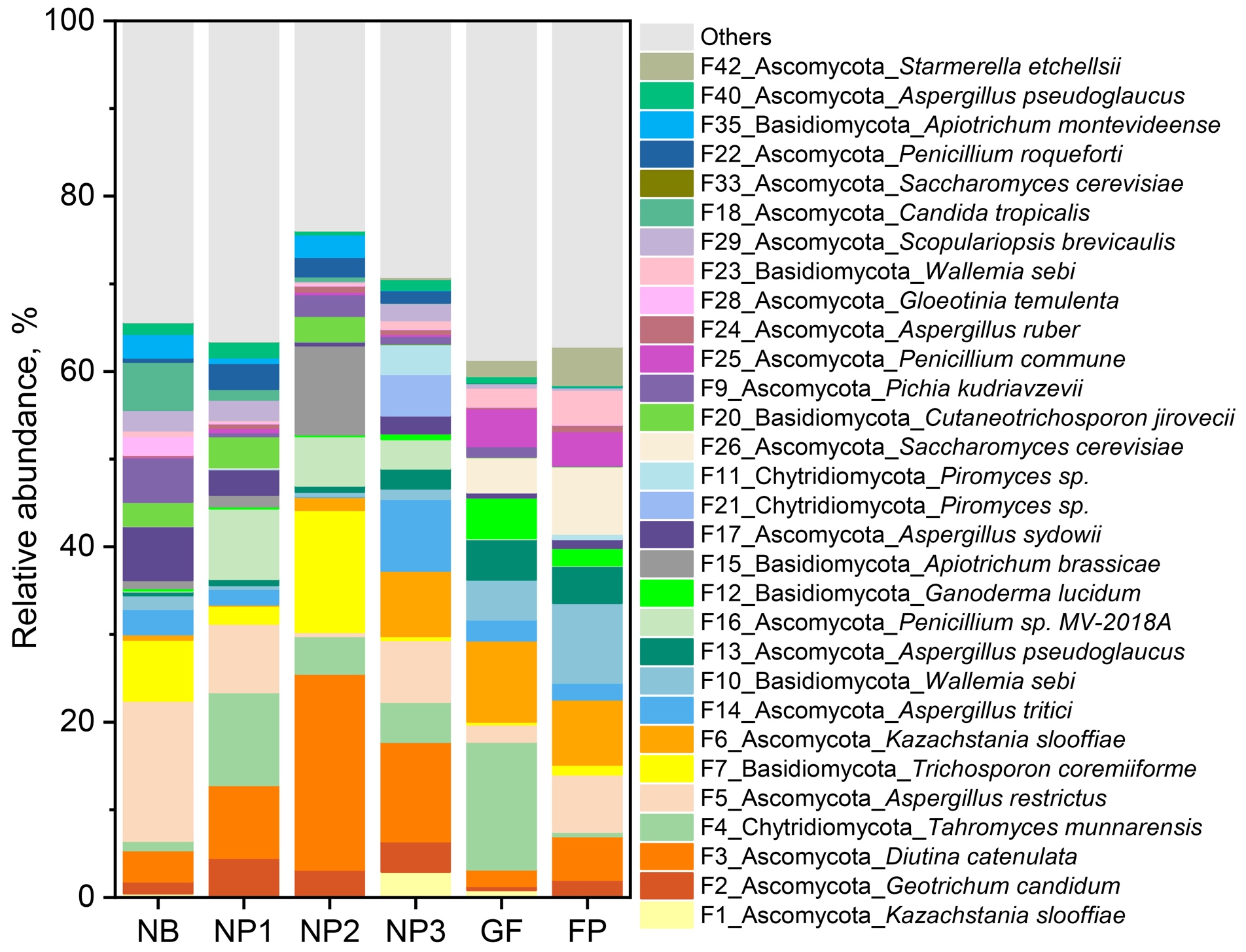


**Fig. S2** Top 30 fungi in different stages of pigs.


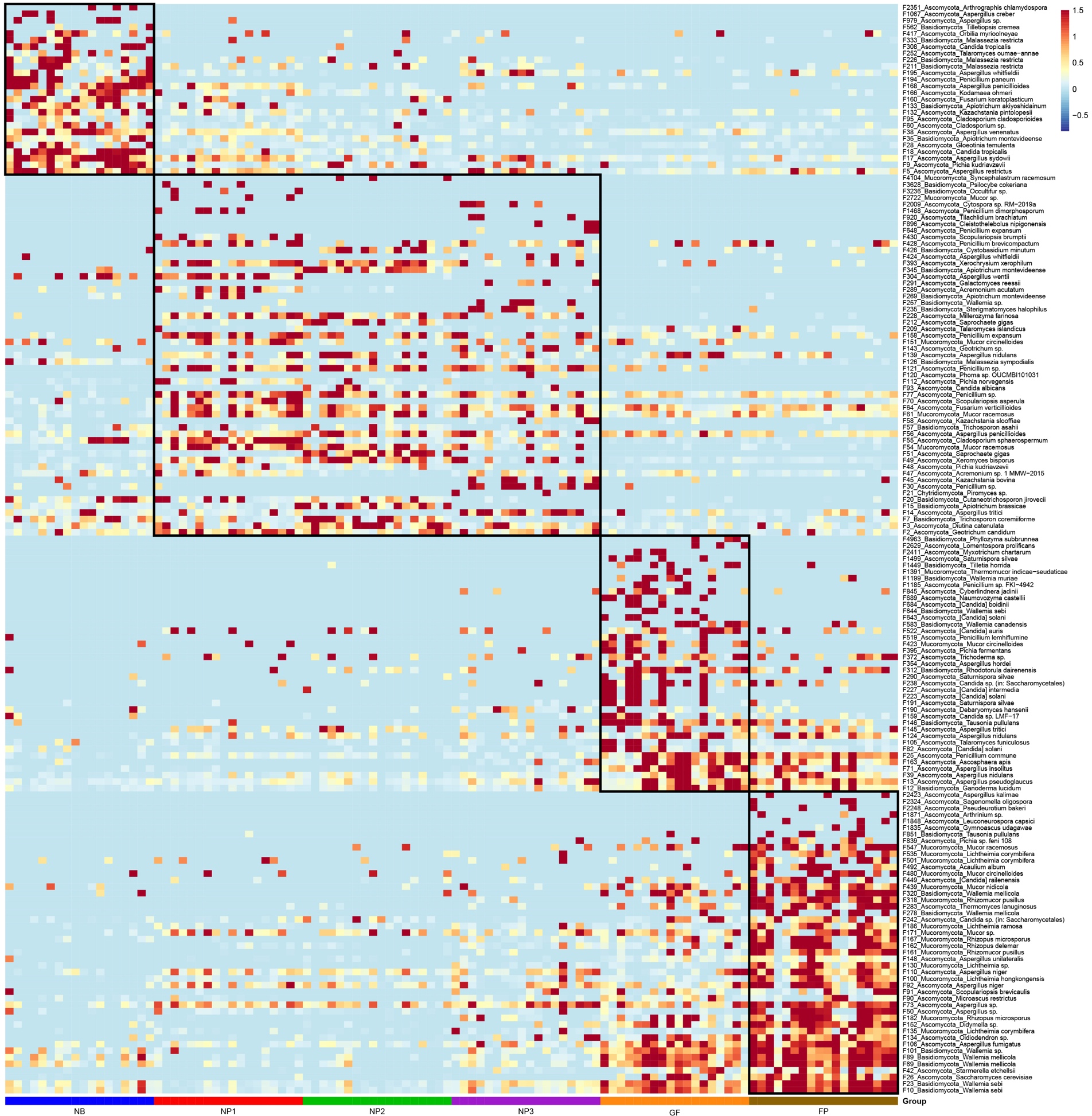


**Fig. S3** Heatmap of stage-related fungi (LDA > 2.0). *NB* lactation piglets (3 d), *NP1* Nursery piglets (26 d), *NP2* Nursery piglets (35 d), *NP3* Nursery piglets (49 d), *GF* Growing pigs (120 d), *FP* Finishing pigs (180 d).


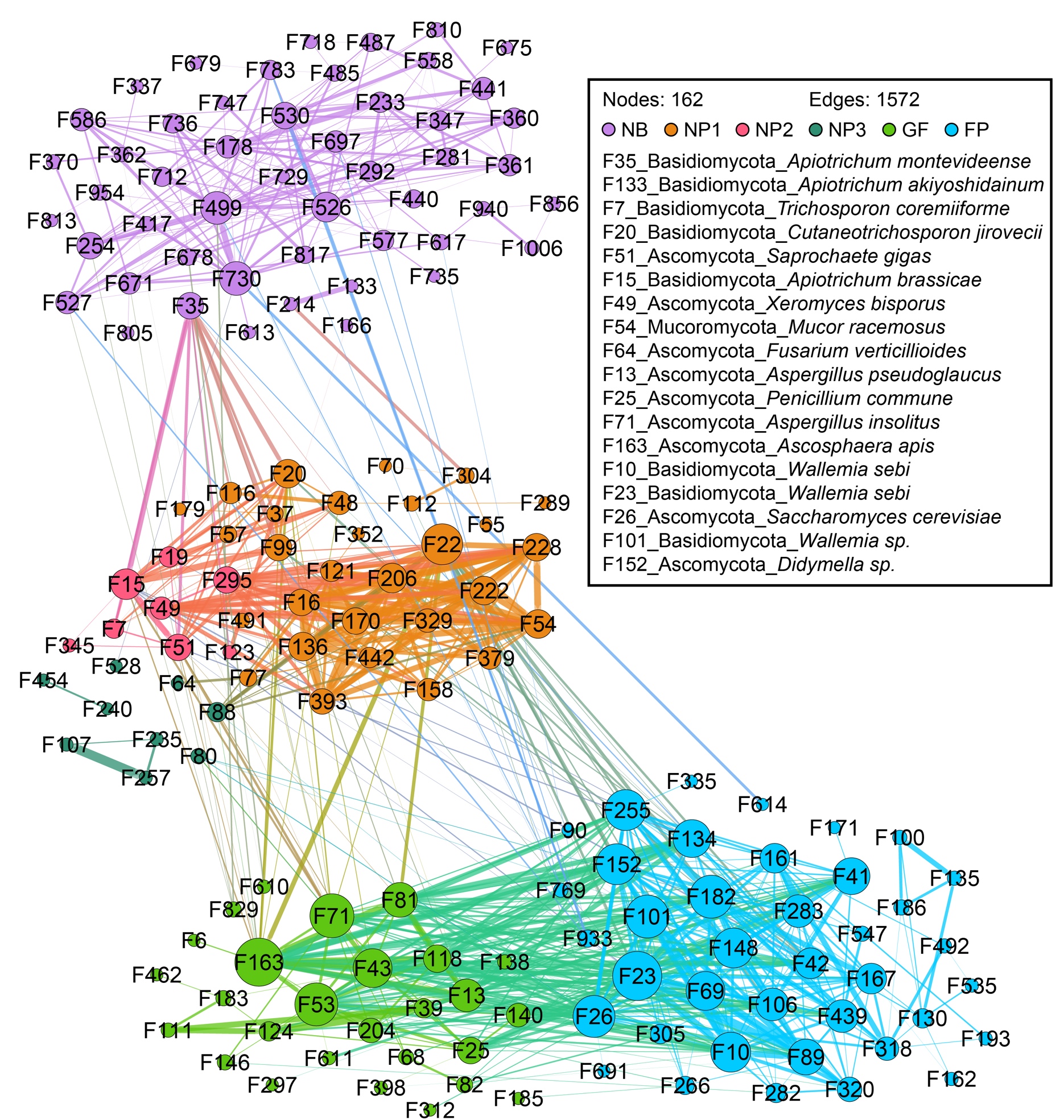


**Fig. S4** Network analysis of interactions at different growth stages. Spearman is used to calculate the top 600 fungi. *NB* lactation piglets (3 d), *NP1* Nursery piglets (26 d), *NP2* Nursery piglets (35 d), *NP3* Nursery piglets (49 d), *GF* Growing pigs (120 d), *FP* Finishing pigs (180 d).


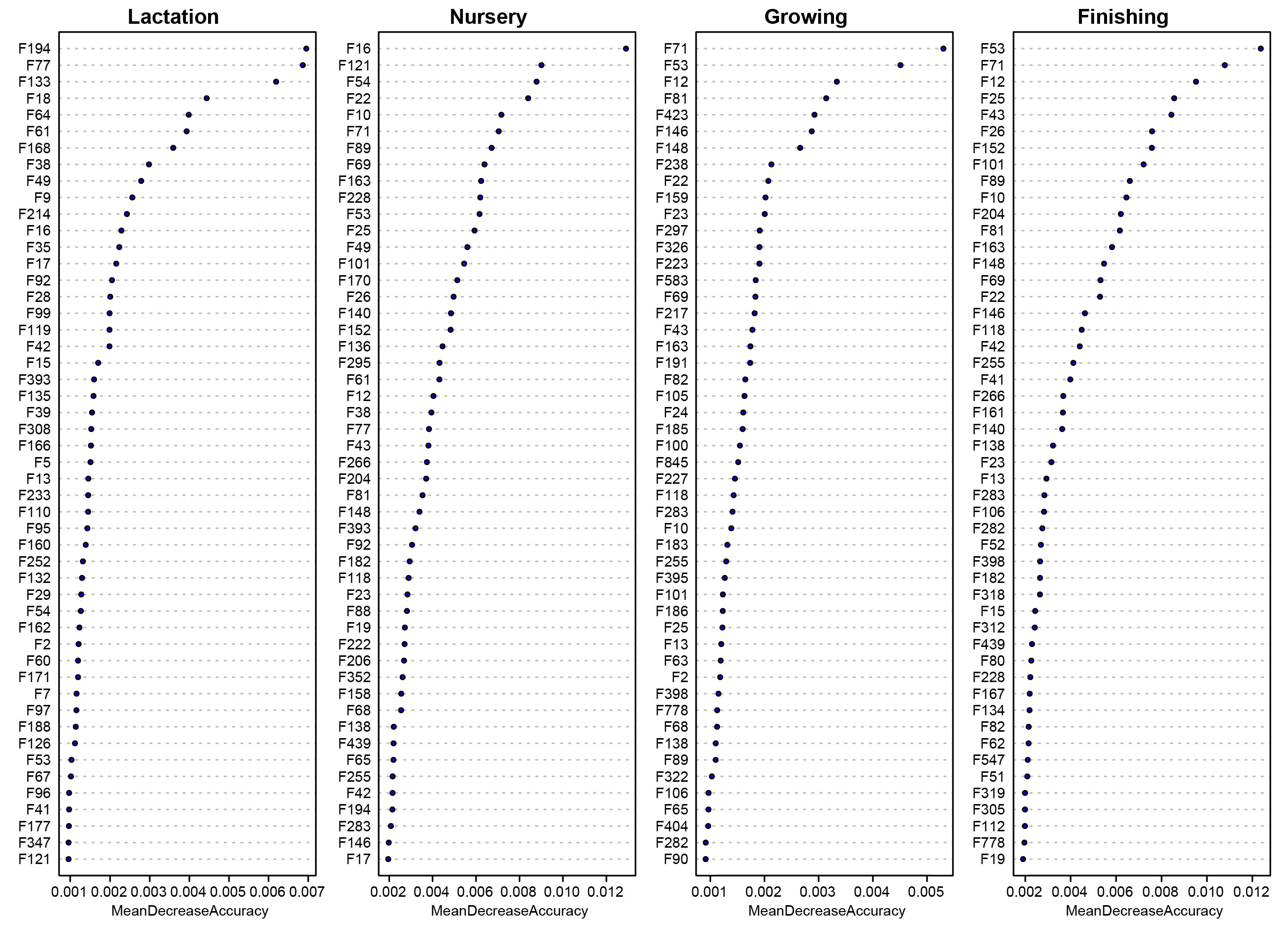


**Fig. S5** Regression-based random forest algorithm is used to select the top 50 growth-related fungi from the top 600 fungi.


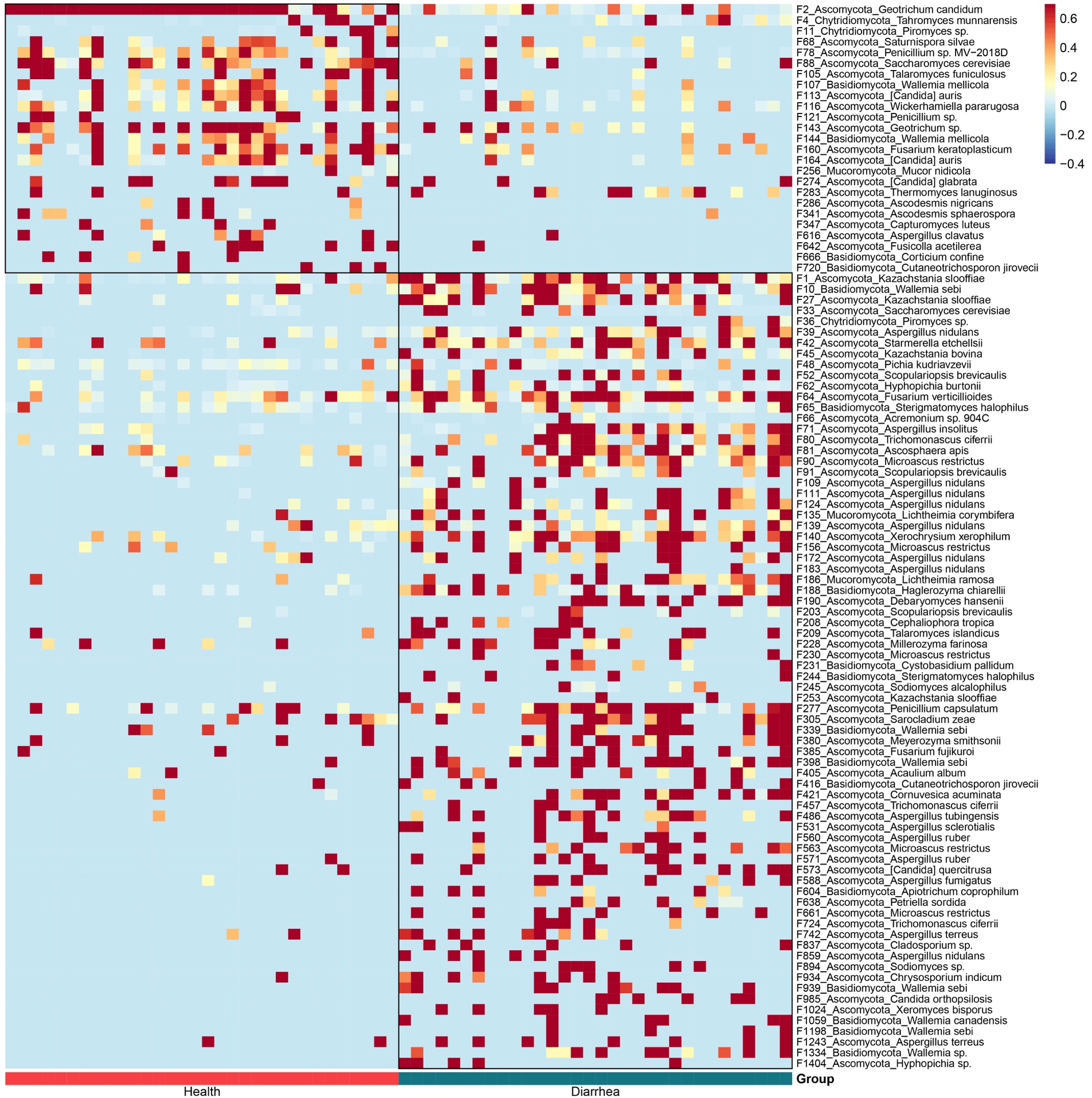
**Fig. S6** Heatmap shows 99 fungi identified by LEfSe in healthy and diarrheal piglets (LDA > 2.0). The top 500 relative abundances were used for LEfSe analysis.
